# Awareness of erosive tooth wear and the consumption of acidic beverages among a group of Finnish adolescents

**DOI:** 10.2340/aos.v84.44568

**Published:** 2025-09-02

**Authors:** Elisa Lind, Auli Suominen, Vuokko Loimaranta, Marja Pöllänen, Merja Laine

**Affiliations:** a Institute of Dentistry, University of Turku, Turku, Finland; b The Finnish Medical Society Duodecim, Current Care, Helsinki, Finland

**Keywords:** erosive tooth wear, adolescent, beverage consumption, knowledge, awareness

## Abstract

**Objective:**

Adolescents belong to the high-risk group of erosive tooth wear (ETW), but by contrast, they are reported to lack knowledge about this condition and its association with the intake of acidic beverages. This study aimed to **survey** the awareness of ETW and the consumption of beverages among a group of Finnish adolescents.

**Material and methods:**

A survey was conducted using an online questionnaire for the students in two upper comprehensive schools in the province of Southwest Finland. The awareness of ETW and the consumption frequencies of 14 beverages according to gender and school grade were analysed using chi-square or Fisher’s exact test. The associations of the awareness of ETW with gender, school grade and acidic beverage consumption were analysed by logistic regression.

**Results:**

Most of the 230 respondents consumed acidic beverages more than once a week. The consumption of lemonades (OR [odds ratio] 2.79–3.05, *p* = 0.008–0.018, 95% CI 1.19–7.00) and energy drinks (OR 5.59, *p* = 0.0008, 95% CI 2.06–15.2) was more common among those who assumed that ETW is not preventable. However, 45–47% of the respondents have not received information about ETW or its prevention from dental care professionals.

**Conclusion:**

The studied Finnish adolescents have a lack of knowledge about ETW and the acidic beverage consumption among them requires intervention.

## Introduction

Dentists working in the Nordic countries are concerned about the increasing prevalence of erosive tooth wear (ETW) [1– 4], which is defined as the combined effects of mechanical wear and chemical dissolution of a tooth surface [5]. This dissolution of tooth surface minerals results from acids of non-bacterial origin. The amount of mineral loss is modulated by several factors such as the duration and frequency of acid exposure, the type of acid involved and the buffering capacity of saliva. The critical pH is defined as the threshold at which equilibrium exists between mineral dissolution and precipitation, and for enamel, it is generally accepted to be 5.5. In contrast, demineralisation in dentin starts at a higher pH, rendering it more susceptible to dissolution compared to enamel. Demineralisation initiates when the pH of the oral cavity decreases below the critical pH [6]. The permanent loss of tooth material can lead to aesthetic concerns **and** functional issues, **such as tooth hypersensitivity** and discomfort or difficulty **during** chewing [7, 8]. The condition has become a common oral health problem, especially among adolescents [9], and the estimated global prevalence of ETW among 12–19-year-olds is 30.4% [10]. By contrast, in Finland, ETW is even more common. The rate of Finnish adolescents with ETW lesions can vary from 67% [11] to as high as 85% [12]. Correspondingly, the ETW prevalence among a group of Finnish conscripts was 62% [13] and among Finnish middle-aged adults 75% [14, 15]. In these studies, ETW was rated from initial to severe using the Basic Erosive Wear Examination (BEWE) index. In most cases, ETW varied from initial to moderate, and the prevalence of severe ETW was rare.

Although ETW can stem from intrinsic factors such as reflux disease or **eating disorders like bulimia**, the significance of acidic drink consumption for ETW deformation is still indisputable [9, 16]. The average pH of juices ranges between 2.3 and 4.7, soft drinks between 2.3 and 3.9 and energy drinks between 2.5 and 4.0, which are all lower than the critical pH for enamel demineralisation [17, 18]. The augmented intake of these kinds of drinks is typical among adolescents in European countries [9, 19–21] as well as in Finland [11, 12, 22, 23]. Dietary education is a part of dental examinations, to which all Finnish adolescents are regularly called. Moreover, health literacy skills, including nutrition education and national dietary guidelines, are taught in the health education subject, which is a compulsory part of the curriculum for graders 7–9. Consequently, both the Finnish National Agency for Education and the Finnish Institute for Health and Welfare have imposed restrictions on the sales of sweetened juices and soft drinks in public schools, which seemed to be a promising way to attenuate adolescents’ consumption of these drinks [24, 25]. Furthermore, the taxation on soft drinks is valid in Finland. However, despite these implementations, the total sales of soft drinks in Finland have increased by 37% within 8 years [26].

The restorative treatment of ETW can be expensive and time consuming, as well as ineffective, without an adequate intervention in the individual risk factors. **Since the** exogenous risk factors are modifiable, their management is a major component of contemporary care of ETW [27]. However, a growing body of literature recognises adolescents’ lack of knowledge about ETW and its association with the intake of acidic drinks [20, 28–31]. Therefore, the present study intends to **survey** the awareness of ETW and the consumption of beverages among a group of Finnish adolescents. **The main hypothesis is that while frequent intake of acidic beverage is common among adolescents, the awareness of ETW remains low.**

## Methods

### Participants

The survey was conducted in autumn 2021 as an electronic questionnaire targeting students from two upper comprehensive schools in Southwest Finland: Turku Lyseo School and Masku Hemminki School. The questionnaire was available for responses between October 25 and November 30. Southwest Finland is predominantly urban, with densely populated areas such as Turku serving as hubs for industry, services and education. The educational level of the population in Southwest Finland is close to the national average. Approximately 13–15% of the province’s population has an immigrant background, which is slightly higher compared to the municipalities in northern Finland. Turku, the sixth largest municipality in Finland, had approximately 195,000 residents in 2021, of whom 12.5% were under the age of 15 years. Masku, located 20 km from Turku, had 9,600 residents, with 19.6% under the age of 15 years. Both participating schools are public institutions for grades 7–9, serving students aged 13–15 years, and are socioeconomically comparable. All students at the schools were invited to participate in the survey. The number of invitations was 186 for Turku Lyseo School and 402 for Masku Hemminki School. Before implementation, the questionnaire was validated for verifying the structure and accessibility using a group of Finnish adolescents (two boys aged 14 and 17 years and two girls aged 15 years), who were not part of the final sample. The required changes were made based on their feedback. The responses of this test group were deleted before the study was officially implemented. An invitation to the study and a Google Forms-based questionnaire were sent to the students using the Wilma pupil administration system. Anonymity was ensured by Google Forms. The questionnaire could be completed using a smartphone, tablet or computer. The partaking adolescents and their guardians were comprehensively informed of the survey and its objectives on an electric form. The school principals gave school-level approval. At Turku Lyseo School, students completed the survey during their physical education or health education classes while the respective teacher was present. The respondents from Masku Hemminki school answered the questions independently during their free time. The presence of a guardian was not required. Participation was voluntary, and direct partaking of the survey was taken as consent for taking part in the study. According to the Ethics Committee of the Hospital District of Southwest Finland, ethical approval was not needed since the collected information was neither of sensitive in nature nor combinable to the individual respondents. After a month without any new responses, despite sending two reminders, the raw data were transferred from Google Forms as an Excel file into the statistical software JMP Pro Version 16.1.0 SAS Institute Inc., Cary, NC.

### The questionnaire

The questionnaire was modified from a previous study made and validated in Norway [20]. The English translation of our questionnaire is available as supplementary data. In the questionnaire, the term ETW was substituted with the word ‘tooth erosion’. The questionnaire held nine statements about ETW to allow further evaluation of the awareness and beliefs of this condition. For statements 1–5, the answer options were given as a five-point Likert scale: totally agree, somewhat agree, I don’t know, somewhat disagree and totally disagree. The answer options ‘yes’, ‘no’ and ‘I don’t know’ were given for statements 6–9. **The Cronbach’s alpha varied between 0.637 and 0.751, and the total consistency for all the statements was 0.739.** The respondents were requested to judge their consumption of 14 beverages, including both acidic and neutral drinks, to the frequency categories: never/rarely, once a week, several times a week, once a day, every day with a meal and every day along the day. Variables such as gender and school grade were elicited to describe the sample and to use as grouping background variables. The consistency of the awareness statements was acceptable to indicate the reliability.

**The questionnaire was completed by 230 students of whom 111 (48.3%) were females and 108 (47.0%) were males. The number of nonbinary persons was 11 (4.8%), and 4 (36.3%) of them were eighth graders and seven (63.6%) were ninth graders. The response rate at Masku Hemminki School was 19.4%, with 78 of 402 invited participants completing the survey. Conversely, Turku Lyseo School had a response rate of 81.7%, with 152 of 186 invited participants responding.**

**The effect size and statistical power for a cross-sectional two-sample study were calculated using R 4.4.1 (package pwr, function pwr.chisq.test). The effect size (*w*) was determined based on the frequencies reported in** Table 1 **for the variable ‘Dental staff have told me about ETW’ stratified by gender. A post hoc power analysis was conducted using the total sample size of 219, an alpha level of 0.05 and an effect size of** ***w* = 0.12, yielding a corresponding statistical power of 33%.**

**Table 1:** The awareness of ETW by gender, school grade and total intake of juice, lemonade and energy drinks (TI) as counts (n) and percentages (%), respectively.

Variables		Total	Gender			School grade				TI consumption frequency				
			Female	Male	*p*-value	7th	8th	9th	*p*-value	Never or rarely	Once a week	Several times a week	Daily	*p*-value
		*n* (%)	*n* (%)	*n* (%)		*n* (%)	*n* (%)	*n* (%)		*n* (%)	*n* (%)	*n* (%)	*n* (%)	
Gender	Female Male	219 111 (50.7) 108 (49.3)				78 (35.6) 31 (27.9) 47 (43.5)	72 (32.9) 36 (32.4) 36 (33.3)	69 (31.5) 44 (39.6) 25 (23.1)		17 (7.8) 8 (7.2) 9 (8.3)	54 (24.7) 33 (29.7) 21 (19.4)	79 (36.1) 37 (33.3) 42 (38.9)	69 (31.5) 33 (29.7) 36 (33.3)	0.369*
School grade	7th gr 8th gr 9th gr	230 78 (33.9) 76 (33.0) 76 (33.0)	111 (48.3)	108 (47.0)						17 (7.4) 7 (9.0) 7 (9.2) 3 (4.0)	55 (23.9) 20 (25.6) 21 (27.6) 14 (18.4)	86 (37.4) 24 (30.8) 26 (34.2) 36 (47.4)	72 (31.3) 27 (34.6) 22 (29.0) 23 (30.3)	0.315*
1. ETW is not preventable	Agree Disagree I’m not sure	64 (27.8) 70 (30.4) 96 (41.7)	30 (27.0) 39 (35.1) 42 (37.8)	30 (27.8) 27 (25.0) 51 (47.2)	0.220*	32 (41.0) 15 (19.2) 31 (39.7)	16 (21.5) 21 (27.6) 39 (51.3)	16 (21.1) 34 (44.7) 26 (34.2)	0.001*	3 (4.7) 3 (4.3) 11 (11.5)	17 (26.6) 20 (28.6) 18 (18.8)	23 (35.9) 28 (40) 35 (36.5)	21 (32.8) 19 (27.1) 32 (33.3)	0.387*
2. ETW can be treated by dentist	Agree Disagree I’m not sure	124 (53.9) 14 (6.1) 92 (40.0)	66 (59.5) 8 (7.2) 37 (33.3)	52 (48.2) 6 (5.6) 50 (46.3)	0.145*	47 (60.3) 5 (6.4) 26 (33.3)	33 (43.4) 3 (4.0) 40 (52.6)	44 (57.9) 6 (7.9) 26 (34.2)	0.096**	6 (4.8) 0 11 (12.0)	35 (28.2) 1 (7.1) 19 (20.7)	50 (40.3) 5 (35.7) 31 (33.7)	33 (26.6) 8 (57.1) 31 (33.7)	0.075**
3. ETW can be prevented by sipping	Agree Disagree I’m not sure	66 (28.7) 42 (18.3) 122 (53.0)	33 (29.7) 21 (18.9) 57 (51.4)	30 (27.8) 18 (16.7) 60 (55.6)	0.815*	29 (37.2) 12 (15.4) 37 (47.4)	15 (19.7) 12 (15.8) 49 (64.5)	22 (29.0) 18 (23.7) 36 (47.4)	0.071*	4 (6.1) 1 (2.4) 12 (9.8)	17 (25.8) 14 (33.3) 24 (19.7)	25 (37.9) 15 (35.7) 46 (37.7)	20 (30.3) 12 (28.6) 40 (32.8)	0.457*
4. ETW can be prevented by tooth brushing	Agree Disagree I’m not sure	126 (54.8) 16 (7.0) 88 (38.3)	62 (55.9) 9 (8.1) 40 (36.0)	56 (51.9) 6 (5.6) 46 (42.6)	0.527*	43 (55.1) 7 (9.0) 28 (35.9)	41 (54.0) 3 (4.0) 32 (42.1)	42 (55.3) 6 (7.9) 28 (36.8)	0.710*	6 (4.8) 1 (6.3) 10 (11.4)	36 (28.6) 2 (12.5) 17 (19.3)	48 (38.1) 5 (31.3) 33 (37.5)	36 (28.6) 8 (50.0) 28 (31.8)	0.240*
5. I want to prevent ETW	Agree Disagree I’m not sure	146 (63.5) 12 (5.2) 72 (31.1)	79 (71.2) 6 (5.4) 26 (23.4)	61 (56.5) 5 (4.6) 42 (38.9)	0.046*	52 (66.7) 6 (7.7) 20 (25.6)	44 (57.9) 0 (0.0) 32 (42.1)	50 (65.8) 6 (7.9) 20 (26.3)	0.016**	8 (5.5) 1 (8.3) 8 (11.1)	43 (29.5) 0 12 (16.7)	60 (41.1) 3 (25.0) 23 (31.9)	35 (24.0) 8 (66.7) 29 (40.3)	0.003**
6. Dental staff have told me about ETW	Agree Disagree I’m not sure	40 (17.4) 108 (47.0) 82 (35.7)	15 (13.5) 64 (57.7) 32 (28.8)	23 (21.3) 41 (38.0) 44 (40.7)	0.013*	13 (16.7) 38 (48.7) 27 (34.6)	10 (13.2) 32 (42.1) 34 (44.7)	17 (22.4) 38 (50.0) 21 (27.6)	0.231*	4 (10.0) 8 (7.4) 5 (6.1)	6 (15.0) 32 (29.6) 17 (20.7)	17 (42.5) 42 (38.9) 27 (32.9)	13 (32.5) 26 (24.1) 33 (40.2)	0.205*
7. I have heard about ETW elsewhere e.g. social media	Agree Disagree I’m not sure	59 (25.7) 104 (45.2) 67 (29.1)	28 (25.2) 55 (49.6) 28 (25.2)	28 (25.9) 46 (42.6) 34 (31.5)	0.511*	16 (20.5) 38 (48.7) 24 (30.8)	18 (23.7) 33 (43.4) 25 (32.9)	25 (32.9) 33 (43.4) 18 (23.7)	0.409*	4 (6.8) 8 (7.7) 5 (7.5)	10 (17.0) 34 (32.7) 11 (16.4)	24 (40.7) 34 (32.7) 28 (41.8)	21 (35.6) 28 (26.9) 23 (34.3)	0.205*
8. I have diagnosed with ETW	Agree Disagree I’m not sure	21 (9.1) 139 (60.4) 70 (30.4)	4 (3.6) 78 (70.3) 29 (26.1)	16 (14.8) 57 (52.8) 35 (32.4)	0.003*	11 (14.1) 43 (55.1) 24 (30.8)	3 (4.0) 44 (57.9) 29 (38.2)	7 (9.2) 52 (68.4) 17 (22.4)	0.060*	2 (9.5) 9 (6.5) 6 (8.6)	2 (9.5) 42 (30.2) 11 (15.7)	6 (28.6) 54 (38.9) 26 (37.1)	11 (52.4) 34 (24.5) 27 (38.6)	0.036*
9. I have received instructions about preventing ETW	Agree Disagree I’m not sure	45 (19.6) 104 (45.2) 81 (35.2)	17 (15.3) 65 (58.6) 29 (26.1)	26 (24.1) 36 (33.3) 46 (42.6)	0.001*	17 (21.8) 36 (46.2) 25 (32.1)	12 (15.8) 29 (38.2) 35 (46.1)	16 (21.1) 39 (51.3) 21 (27.6)	0.182*	3 (6.7) 8 (7.7) 6 (7.4)	10 (22.2) 31 (29.8) 14 (17.3)	17 (37.8) 39 (37.5) 30 (37.0)	15 (33.3) 26 (25.0) 31 (38.3)	0.446*

Nonbinary persons (*n* = 11) are excluded from the gender-based analysis.

* Chi square,

** Fisher’s exact test. ETW: erosive tooth wear.

### Statistical analysis

The statistical analysis was performed using JMP^®^ Pro Version 16.1.0 SAS Institute Inc., Cary, NC. The statistical significance was considered at *p* < 0.05. The categorical variables were summarised with counts and percentages. The statements concerning the awareness of ETW with Likert scale options (1–5) ‘totally agree’ and ‘somewhat agree’ were coalesced into ‘agree’ and correspondingly ‘totally disagree’ and ‘somewhat disagree’ into ‘disagree’. The consistency of the statements with the Likert scale was calculated using Cronbach’s alpha. The studied 14 beverages were categorised into the following groups: neutral drinks (water, plain or flavoured mineral water, milk), coffee and tea, ice tea and kombucha, juice (ready-to-drink and self-made juice), lemonade (normal and light) and energy drinks. The intake of juice, lemonade and energy drinks was combined into the group ‘total intake’ (TI). For the consumption frequency analysis, the responses ‘once a day’, ‘every day with a meal’ and ‘every day along the day’ were pooled as ‘daily’ use. Other answer options were ‘several times a week’, ‘once a week’ and ‘never or rarely’. The more frequent use categories were compared to the lower ones. Due to the small number of respondents (*n* = 11), nonbinary persons were excluded from the gender-based analysis but included in the school grade-based analysis. The association analyses between gender (*n* = 219) and school grade (*n* = 230) for both the consumption of the drinks and the statements about ETW were analysed with the chi-square test. If the assumptions were not valid, the analysis was performed with Fisher’s exact test.

For further analyses, the acidic beverage consumption was dichotomised: no more than once a week and at least several times weekly. First, the consumption of lemonade, energy drinks and TI (dependent) was analysed by univariate logistic regression with gender, school grade and awareness statements (independent). The included statements presented statistical significance in the bivariate analysis. Second, the multivariate model elaborated on whether the connection between beverage consumption (dependent) and the awareness of erosion (independent) differed between gender or school grade. Due to multicollinearity between the awareness of ETW, three stable models were built up, including one of the statements. ‘Dental staff have told me about tooth erosion’ (Model 1) was used to study risk factors for energy drink consumption. ‘I have received instructions about preventing tooth erosion’ (Model 2) was used for TI. The associations with lemonade consumption were studied with the statement ‘I have been diagnosed with tooth erosion’ (Model 3) and the statements from models 1 and 2. All the interactions between the statements, gender and school grade were studied, and the statistically significant interactions were included in the models. The odds ratio (OR) and 95% confidence intervals (95% CI) with *p*-values were calculated.

## Results

The results obtained from the association analyses between the ETW statements and TI are summarised in Table 1. Over half of those surveyed reported that ETW can be prevented by tooth brushing or treated by a dentist. Only 18% knew that ETW cannot be avoided by sipping a beverage. These knowledge questions did not statistically differ between the genders although males have received more information about ETW or its preventing strategies compared to the females (*p* = 0.013 and *p* = 0.001, respectively). **Approximately, 30%** of our respondents assumed that the development of ETW is not preventable. The respondents who agreed with this opinion consumed three times more likely lemonades and five times more likely energy drinks several times a week than the ones who thought that ETW is preventable.

The results of the binary analysis of beverage consumption are provided as supplementary data. The results of the logistic regression analyses of the consumption of lemonade, energy drinks and TI are set out in Tables 2 and 3. There was one statistically significant interaction in the multivariate model 3, which compared the lemonade consumption frequencies ‘several times a week or daily’ to ‘once a week or less’ (Table 2). It was between the variables ‘gender’ and the statement ‘I have been diagnosed with ETW’ (*p* = 0.045). Consequently, in this model, male gender did not remain significant contrary to models 1 and 2 (OR 1.93 *p* = 0.033, 95% CI 1.05–3.53 and OR 1.92, *p* = 0.037, 95% CI 1.04–3.55, respectively). However, the opinion that ETW is not preventable remained significant in all three models (*p* = 0.008–0.018). Compared to the respondents who consume lemonade once or less a week, the probability of agreement with this opinion was increased among those who consume lemonade several times a week or daily (OR 2.79–3.05, 95% CI 1.19–7.00).

**Table 2:** The associations of the consumption of lemonade (several times a week or daily = 1) with the confounding variables gender, school grade and awareness of ETW by logistic regression.

Variables		Lemonade											
		Univariate model			Multivariate model 1			Multivariate model 2			Multivariate model 3		
		OR	*p*-value	Confidence interval 95%	OR	*p*-value	Confidence interval 95%	OR	*p*-value	Confidence interval 95%	OR	*p*-value	Confidence interval 95%
Gender	Girls (ref) Boys	1.88	0.025	1.08–3.27	1.93	0.033	1.05–3.53	1.92	0.037	1.04–3.55	1.48	0.387	0.61–3.58
School grade	7th gr 8th gr (ref) 9th gr 9th vs 7th	0.94 1.36 1.45	0.848 0.372 0.273	0.48–1.83 0.69–2.68 0.75–2.83	0.72 1.66 2.29	0.384 0.181 0.031	0.35–1.50 0.79–3.48 1.08–4.87	0.69 1.57 2.28	0.315 0.238 0.033	0.33–1.43 0.74–3.29 1.07–4.86	0.75 2.06 2.73	0.459 0.071 0.012	0.36–1.59 0.94–4.50 1.25–5.96
ETW is not preventable	Disagree (ref) Agree I’m not sure	2.18 1.84	0.040 0.079	1.04–4.59 0.93–3.64	3.04 2.10	0.008 0.102	1.33–6.93 0.86–5.12	3.05 2.17	0.008 0.087	1.33–7.00 0.89–5.26	2.79 2.02	0.018 0.121	1.19–6.56 0.83–4.94
I want to prevent ETW	Disagree (ref) Agree I’m not sure	0.42 0.66	0.171 0.521	0.12–1.45 0.18–2.37	0.29 0.38	0.074 0.214	0.08–1.13 0.08–1.74	0.27 0.40	0.066 0.241	0.07–1.07 0.09–1.84	0.29 0.39	0.083 0.237	0.07–1.17 0.09–1.84
Dental staff have told me about ETW	Disagree (ref) Agree I’m not sure	1.55 1.25	0.255 0.475	0.73–3.30 0.68–2.30	1.37 1.09	0.460 0.793	0.59–3.18 0.57–2.10						
I have received instructions about preventing ETW	Disagree (ref) Agree I’m not sure	1.80 1.06	0.112 0.853	0.87–3.72 0.57–1.98				1.74 0.85	0.173 0.656	0.78–3.88 0.43–1.71			
I have diagnosed with ETW	Disagree (ref) Agree I’m not sure	2.53 1.61	0.056 0.127	0.98–6.55 0.87–2.96							1.96 1.54	0.285 0.210	0.57–6.74 0.78–3.03

Nonbinary persons (*n* = 11) are excluded from the analysis. ETW: erosive tooth wear; OR: odds ratio.

**Table 3:** The associations of the consumption of energy drinks (several times a week or daily = 1) and the associations of total intake of acidic beverages (several times a week or daily = 1) with the confounding variables gender, school grade and awareness of ETW by logistic regression.

Variables		Energy drinks						Total intake of juice, lemonade and energy drinks					
		Univariate model			Multivariate model 1			Univariate model			Multivariate model 2		
		OR	*p*-value	Confidence interval 95%	OR	*p*-value	Confidence interval 95%	OR	*p*-value	Confidence interval 95%	OR	*p*-value	Confidence interval 95%
Gender	Female (ref) Male	1.25	0.463	0.69–2.29	1.23	0.552	0.62–2.45	1.52	0.149	0.86–2.70	1.56	0.160	0.84–2.92
School grade	7th gr 8th gr (ref) 9th gr 9th vs 7th	2.58 4.78 1.85	0.031 0.0003 0.081	1.09–6.09 2.05–11.2 0.93–3.71	2.18 7.16 3.28	0.093 <0.0001 0.004	0.88–5.42 2.80–18.3 1.46–7.38	1.20 2.10 1.75	0.588 0.046 0.131	0.62–2.34 1.01–4.39 0.85–3.63	1.07 2.57 2.38	0.836 0.019 0.035	0.53–2.20 1.17–5.64 1.06–5.32
ETW is not preventable	Disagree (ref) Agree I’m not sure	3.24 2.17	0.007 0.061	1.38–7.61 0.97–4.89	5.59 4.04	0.0008 0.0120	2.06–15.2 1.36–12.0	1.15 1.18	0.705 0.628	0.55–2.43 0.60–2.31	1.56 1.42	0.282 0.429	0.69–3.50 0.59–3.40
I want to prevent ETW	Disagree (ref) Agree I’m not sure	0.58 0.68	0.412 0.570	0.16–2.11 0.18–2.59	0.47 0.48	0.304 0.373	0.11–1.99 0.09–2.45	0.18 0.24	0.107 0.187	0.02–1.45 0.03–2.00	0.19 0.21	0.129 0.185	0.02–1.62 0.02–2.08
Dental staff have told me about ETW	Disagree (ref) Agree I’m not sure	1.76 1.29	0.174 0.464	0.78–3.95 0.65–2.54	1.75	0.245	0.68–4.46	1.65 1.45	0.231 0.251	0.73–3.78 0.77–2.74			
I have received instructions about preventing ETW	Disagree (ref) Agree I’m not sure	1.84 1.21	0.1171 0.580	0.86–3.94 0.61–2.41				1.39 1.66	0.397 0.128	0.65–2.99 0.86–3.18	1.27 1.26	0.560 0.611	0.56–2.91 0.51–3.12
I have diagnosed with ETW	Disagree (ref) Agree I’m not sure	3.35 1.34	0.014 0.3819	1.28–8.80 0.70–2.57				2.35 1.63	0.145 0.146	0.75–7.43 0.84–3.13			

Nonbinary persons (*n* = 11) are excluded from the analysis. ETW: erosive tooth wear; OR: odds ratio.

In the multivariate analysis concerning energy drink consumption or TI (Table 3), there were no statistically significant interactions. Including all the variables of model 1, only ‘ninth vs eighth grade’ (OR 7.16, *p* <0.0001, 95% CI 2.80–18.3) and the opinion that ‘ETW is not preventable’ (OR 5.59, *p* = 0.0008, 95% CI 2.06–15.2) remained statistically significantly associated with a higher energy drink consumption. The corresponding remaining significance of TI in model 2 was ‘ninth vs eighth grade’ (OR 2.57, *p* = 0.019, 95% CI 1.17–5.64).

## Discussion

The present study was designed to determine the awareness and attitudes of ETW and the beverage consumption among the group of Finnish adolescents. Despite the increasing prevalence of ETW, only a limited number of Finnish ETW studies have been conducted, and the research has tended to focus on prevalence rather than awareness.

**The present study indicates that the studied group of Finnish adolescents lack knowledge about ETW and its association with acidic drink intake, thus supporting the hypothesis.** This is consistent with the earlier studies [20, 28–31]. This is **particularly concerning, as** public dental care **in Finland** is free for all minors, and the dental examinations include preventive care and dietary education. Still, a preponderance of our **participants reported not receiving any** information about ETW or its prevention from dental care professionals. Furthermore, only 9% of our **participants reported having been diagnosed with ETW. There is a substantial difference to the prevalence of Finnish adolescents with ETW lesions, which has been discovered to be 67–85%** [11, 12]. However, Skudutyte-Rysstad et al. [30] observed that only half of their studied 18-year-old Norwegians with ETW were aware of this diagnosis; thus the number of respondents with ETW can also be higher among our study population. In the study of Kangasmaa et al. [2], it was established that the awareness of ETW among Finnish dentists is high, and the participated dentists systematically reported ETW findings and dietary histories in patient files. However, it remains unclear whether the adolescents of our study have not received information from oral health care professionals, have forgotten the provided information due to the time elapsed since their last dental examination, received the information in rush or if other factors, such as misunderstanding the question, played a role. It should also be considered that if the information provided was not perceived as relevant to the adolescents’ current concerns or lifestyle, they may have disregarded it. However, encouraging was that the majority of our respondents indicated the will to prevent the development of ETW.

Two-thirds of those surveyed reported consuming juice, soft drinks and/or energy drinks more than once a week as reported earlier in other Finnish studies [11, 12, 22]. The number of adolescents (31%) who consume these drinks daily is in line with the study from Karppinen et al. [12] but on the contrary, higher than (16%) found in the study from Methuen et al. [11]. In the present study, daily use of acidic drinks and male gender were significantly associated with the answer ‘I have been diagnosed with tooth erosion’. In contrast to earlier findings [11, 12, 19–23], our analysis supports the gender-based differences only in the case of high lemonade consumption, which was twice as likely among males than females. Vereecken et al. [32], who compared daily soft drink consumption levels among European adolescents, found that in all but five of the 28 studied countries, boys drank soft drinks significantly more often than girls. On average, 26% of 114,558 adolescents aged 11–15 years reported daily soft drink intake. In the Nordic countries (excluding Iceland), where public health recommendations **emphasise limiting the intake of acidic beverages** due to its damaging effect on dental health, daily consumption rates were among the lowest, ranging from 9 to 20% for 13-years-olds, and from 8 to 26% for 15-year-olds. This lower daily soft drink intake may reflect the broader health-promoting culture in the region, including strict regulations on soft drink advertising and availability, particularly in schools where sugary beverages are often banned, and school meals typically include water and milk. In contrast, **data from Scotland and Wales show higher daily soft drink consumption rates: from 37 to 46% among 13-year-olds and from 38 to 49% among 15-year-olds. The dietary guidelines in the United Kingdom primarily focus on reducing sugar intake rather than explicitly addressing beverage acidity.** Furthermore, in **these regions**, the marketing of soft drinks is strong, and **such beverages** are often available in school vending machines. Similarly, in **Western Europe** (including **France, Belgium, the Netherlands and Switzerland**), daily soft drink consumption was also higher than in the Nordic countries. The corresponding proportions ranged from 29 to 46% among 13-year-olds and from 29 to 49% among 15-year-olds. Although national recommendations and the regulation of soft drink sales in schools are not as strict as in the Nordic countries, advertising still aims to promote healthier choices for adolescents [17, 32].

According to a recent systematic review and meta-analysis, the prevalence for daily energy drink users is in globally 9% (95% CI = 6.3, 11.4), for Europeans 7% (95% CI = 3.5, 10.1) and for adolescents 8% (95% CI = 4.4, 11.2) [33]. Our results are in line with these finding as 10% of our study population consumed energy drinks daily, and 17% consumed them several times a week. Another finding was that the energy drink consumption was twice or even seven times more likely among ninth graders compared to seventh and eighth graders, respectively. This is likely due to the fact that, although there is no specific legislation in Finland regarding the energy drink sale to minors, many retail chains have independently imposed restrictions on the energy drink sale to those under the age of 15. In contrast, in several European countries, such as France and Hungary, the sale of energy drinks to adolescents is more strictly regulated, and in some countries, they cannot be sold to those under 18. In addition, the ninth graders are on the threshold of a transition phase, moving from adolescence towards young adulthood, where parental monitoring is dismissing, and adolescents become more responsible for their diet [34]. Therefore, interventions targeting ninth graders in particular would be advantageous. While altering dietary habits can be challenging, prior research [22, 35] has shown that dietary interventions conducted by dental personnel are an effective method for modifying patients’ dietary behaviour. However, adolescents own personal beliefs and attitudes affect how they process and use the health information. Support from the school system would also be needed to alter adolescents’ attitudes of acidic drink consumption culture and importance of oral health. Currently, oral health is not mentioned in the national health education curriculum [36], and albeit the extensive oral health education material is available, the teaching of it tends to depend on the activity of the teacher in charge [37, 38].

To our knowledge, this study is the first one to **survey** the awareness of ETW among Finnish adolescents and supports the concern about the oral health status of this age group as also highlighted by Methuen et al. [11, 22]. However, the generalisability of these results to a broader population of Finnish adolescents is limited by the fact that the study cohort represents only a small group of Finnish adolescents from one province of Finland. Correspondingly, the relatively low response rate reduced statistical power and may lead to response bias. An explanation for the low response rate (230 of 588 invited) might be that at Masku Hemminki school, responding to the survey depended on the students’ own activity (response rate 19.4%), whereas at Turku Lyseo school a time slot was set aside for this purpose (response rate 81.7%). It is also possible that some intended respondents may have missed the pupil administration system-mediated study invitation, which could affect the sample’s representativeness. Correspondingly, only those who chose to respond were included to the study, which may skew the results and affect the diversity of responses. For future studies, a standardised setting should be set to reduce bias, and more detailed instructions should be provided to the schools. Other sources of uncertainty lie in the nature of a questionnaire study. Self-reports might be more optimistic than reality, and the participants might give considered desirable answers or in contrary, the answers might be unserious or incomplete. In addition, the respondents might estimate their beverage intake according to the present moment instead of the actual total consumption. For example, beverage consumption may differ largely between summer and winter. In a survey like this, the reliability of the responses can also be affected by survey fatigue if the respondents found the questionnaire too long or complex. Whereas an online questionnaire favours this tech-savvy target group, technical issues, like problems with Internet connection or used device, can lead to frustration and incomplete responses. Only 21 respondents reported their positive ETW diagnosis, and in this study, the diagnosis was not ensured by clinical examination. Consequently, the association between inadequate awareness of ETW and the clinical occurrence of this condition needs to be confirmed in further research. It must also be considered that in spite of the term ‘tooth erosion’ was explained at the beginning of the questionnaire, the word ‘tooth erosion’ can be a foreign concept and, at the dentist’s office or on social media, may have substituted with other terms, for example, ‘influence of acids’. In addition, without the possibility to clarify questions in real time, respondents might misinterpret questions or answer incorrectly. Nevertheless, Cronbach’s alpha of the statements indicated high internal consistency of the scale. Another strength of our survey was that distributions between males and females, as well as school grades, were relatively equal.

Taken together, these results, while preliminary, indicate that greater efforts are needed to ensure adolescents’ awareness of ETW, and this topic needs, indeed, to be studied and discussed further. Particularly, the knowledge regarding the role of acidic beverages in the ETW aetiology requires further reinforcement. Consequently, adolescents’ increasingly high acidic drink consumption not only has caused a higher incidence of ETW but also has been associated with growing issues of caries and obesity among this age group [39–41]. For creating a base for future health, interventions to decrease acidic drink consumption should be targeted at all adolescents and should be included in all dental examinations. Furthermore, oral health professionals should enhance the policies in collaboration with education agencies, and greater efforts are needed to ensure the incorporation of oral health into the health education curriculum.

## Conclusion

The present study indicates that Finnish adolescents lack knowledge about ETW and its association with acidic drink intake. Targeted interventions for this age group should be implemented due to increased awareness of ETW and the role of acidic drink consumption in its aetiology because the prevention of ETW is effective and always the best treatment.

## Supplementary Material


