# Supplementary material for: Awareness of erosive tooth wear and the consumption of acidic beverages among a group of Finnish adolescents
Source: Acta Odontol Scand. 2025 Sep 2;84:44568. doi: 10.2340/aos.v84.44568 (PMC12534842; doi:10.2340/aos.v84.44568)
Supplement: Supplementary file 1 [file AOS-84-44568-s1.pdf]

Supplementary material has been published as submitted. It has not been copyedited or typeset by Acta Odontologica Scandinavica.

Consumption habits of non-alcoholic beverages among Finnish upper comprehensive school students

The aim of this electronic survey is to investigate how often Finnish adolescents consume the following commonly available beverages. The study will be published in a dental journal and is part of a dissertation on dental erosion. Participation in the survey is voluntary. Responses cannot be linked to the respondents.

1. Gender
   a. Female
   b. Male
   c. Nonbinary person

2. School
   a. Masku Hemminki school
   b. Turku Lyseo school

3. School grade
   a. 7th
   b. 8th
   c. 9th

4. How often do you drink the following types of beverage? Choose the most appropriate option from the grid.

	**Never or rarely**	**Once a week**	**Several times a week**	**Once a day**	**Every day with a meal**	**Every day along the day**
**Water**						
**Plain mineral water**						
**Flavored mineral water**						
**Milk**						
**Coffee**						
**Tea**						
**Ice tea**						
**Kombucha**						
**Fruit juice**						
**Self-made juice**						
**Ready-to-drink -juice**						
**Lemonade**						
**Light lemonade**						
**Energy drink**						

In tooth erosion, the surface of the tooth dissolves when it comes into contact with acid. The dissolved tooth surface can become sensitive and is prone to wear caused by biting.
Erosion is becoming more common, especially among young adults, and its most common risk factor is the excessive consumption of acidic beverages.

5. What do you know about tooth erosion? Choose the most appropriate option from the grid.

	**Totally agree**	**Somewhat agree**	**I don't know**	**Somewhat disagree**	**Totally disagree**
**Tooth erosion is not preventable**					
**Tooth erosion can be treated by dentist**					
**Tooth erosion can be prevented by sipping**					
**Tooth erosion can be prevented by tooth brushing**					
**I want to prevent tooth erosion**					

6. Have you received information about tooth erosion from your dental clinic? Select the most appropriate option from the grid.

	**Yes**	**No**	**I don't know**
**Dental staff have told me about tooth erosion**			
**I have heard about tooth erosion elsewhere e.g., social media**			
**I have diagnosed with tooth erosion**			
**I have received instructions about preventing tooth erosion**			

Consumption frequencies of the beverages by gender (n=219) and school grade (n=230) as counts (n) and percentages (%), respectively. Nonbinary persons (n=11) are excluded from the gender-based analysis. *Fisher´s Exact Test, **Chi Square.

		Never or rarely<br>n (%)	Once a week<br>n (%)	Several times a week<br>n (%)	Daily<br>n (%)	p-value
**Neutral drinks:** Water, plain or flavored mineral water, milk						
Gender		3 (1.4)	3 (1.4)	15 (6.9)	198 (90.4)	0.882*
	Female n=111	2 (1.8)	1 (0.9)	7 (6.3)	101 (90.1)	
	Male n=108	1 (0.9)	2 (1.9)	8 (7.4)	97 (89.8)	
School grade		3 (1.3)	3 (1.3)	19 (8.3)	205 (89.1)	0.393*
	7th gr. n=78	2 (2.6)	2 (2.6)	6 (7.7)	68 (87.2)	
	8th gr. n=76	1 (1.3)	0	4 (5.3)	71 (93.4)	
	9th gr. n=76	0	1 (1.3)	9 (11.8)	66 (86.8)	
**Coffee and tea**						
Gender		115 (52.5)	30 (13.7)	35 (16.0)	39 (17.8)	0.691**
	Female	58 (52.3)	16 (14.4)	20 (18.0)	17 (15.3)	
	Male	57 (52.8)	14 (13.0)	15 (13.9)	22 (20.4)	
School grade		122 (53.0)	31 (13.5)	38 (16.5)	39 (17.0)	0.591**
	7th gr.	36 (46.2)	10 (12.8)	16 (20.5)	16 (20.5)	
	8th gr.	46 (60.5)	10 (13.2)	11 (14.5)	9 (11.8)	
	9th gr.	40 (52.6)	11 (14.5)	11 (14.5)	14 (18.4)	
**Ice tea and kombucha**						
Gender		168 (76.7)	29 (13.2)	16 (7.3)	6 (2.7)	0.592*
	Female	83 (74.8)	16 (14.4)	10 (9.0)	2 (1.8)	
	Male	85 (78.7)	13 (12.0)	6 (5.6)	4 (3.7)	
School grade		177 (77.0)	29 (12.6)	18 (7.8)	6 (2.6)	0.413*
	7th gr.	54 (69.2)	13 (16.7)	7 (9.0)	4 (5.1)	
	8th gr.	60 (79.0)	10 (13.2)	5 (6.6)	1 (1.3)	
	9th gr.	63 (82.9)	6 (7.9)	6 (7.9)	1 (1.3)	
**Juice:** Ready-to-drink or self-made juice						
Gender		43 (19.6)	68 (31.1)	67 (30.6)	41 (18.7)	0.411**
	Female	22 (19.8)	37 (33.3)	36 (32.4)	16 (14.4)	
	Male	21 (19.4)	31 (28.7)	31 (28.7)	25 (23.2)	
School grade		46 (20.0)	69 (30.0)	73 (31.7)	42 (18.3)	0.841**
	7th gr.	14 (18.0)	22 (28.2)	24 (30.8)	18 (23.1)	
	8th gr.	18 (23.7)	24 (31.6)	23 (30.3)	11 (14.5)	
	9th gr.	14 (18.4)	23 (30.3)	26 (34.2)	13 (17.1)	
**Lemonade:** Normal or light						
Gender		59 (26.9)	77 (35.2)	54 (24.7)	29 (13.2)	0.104**
	Female	36 (32.4)	41 (36.9)	21 (18.9)	13 (11.7)	
	Male	23 (21.3)	36 (33.3)	33 (30.6)	16 (14.8)	
School grade		60 (26.1)	80 (34.8)	59 (25.7)	31 (13.5)	0.715**
	7th gr.	23 (29.5)	28 (35.9)	16 (20.5)	11 (14.1)	
	8th gr.	19 (25.0)	29 (38.2)	18 (23.7)	10 (13.2)	
	9th gr.	18 (23.7)	23 (30.3)	25 (32.9)	10 (13.2)	
**Energy drinks**						
Gender		120 (54.8)	41 (18.7)	37 (16.9)	21 (9.6)	0.159**
	Female	57 (51.4)	27 (24.3)	16 (14.4)	11 (9.9)	
	Male	63 (58.3)	14 (13.0)	21 (19.4)	10 (9.3)	
School grade		124 (53.9)	44 (19.1)	40 (17.4)	22 (9.6)	<0.0001**
	7th gr.	49 (62.8)	8 (10.3)	13 (16.7)	8 (10.3)	
	8th gr.	51 (67.1)	15 (19.7)	5 (6.6)	5 (6.6)	
	9th gr.	24 (31.6)	21 (27.6)	22 (28.9)	9 (11.8)	
